# Automatic 3D reconstruction of vertebrae from orthogonal bi-planar radiographs

**DOI:** 10.1038/s41598-024-65795-7

**Published:** 2024-07-13

**Authors:** Yuepeng Chen, Yue Gao, Xiangling Fu, Yingyin Chen, Ji Wu, Chenyi Guo, Xiaodong Li

**Affiliations:** 1 School of Computer Science (National Pilot Software Engineering School), Beijing University of Posts and Telecommunications, Beijing, 100876 China; 2 Key Laboratory of Trustworthy Distributed Computing and Service (BUPT), Ministry of Education, Beijing, 100876 China; 3 Institute for Intelligent Healthcare, Tsinghua University, Beijing, 100084 China; 4 Guangdong Provincial Key Laboratory of Tumor Interventional Diagnosis and Treatment, Zhuhai People’s Hospital, Zhuhai, 519000 China; 5 Department of Electronic Engineering, Tsinghua University, Beijing, 100084 China; 6 College of AI, Tsinghua University, Beijing, 100084 China; 7 Department of Spine and Osteology, Zhuhai People’s Hospital, Zhuhai, 519000 China

**Keywords:** Biomedical engineering, Radiography, Three-dimensional imaging

## Abstract

When conducting spine-related diagnosis and surgery, the three-dimensional (3D) upright posture of the spine under natural weight bearing is of significant clinical value for physicians to analyze the force on the spine. However, existing medical imaging technologies cannot meet current requirements of medical service. On the one hand, the mainstream 3D volumetric imaging modalities (e.g. CT and MRI) require patients to lie down during the imaging process. On the other hand, the imaging modalities conducted in an upright posture (e.g. radiograph) can only realize 2D projections, which lose the valid information of spinal anatomy and curvature. Developments of deep learning-based 3D reconstruction methods bring potential to overcome the limitations of the existing medical imaging technologies. To deal with the limitations of current medical imaging technologies as is described above, in this paper, we propose a novel deep learning framework, **ReVerteR**, which can realize automatic 3D **Re**construction of **Verte**brae from orthogonal bi-planar **R**adiographs. With the utilization of self-attention mechanism and specially designed loss function combining Dice, Hausdorff, Focal, and MSE, ReVerteR can alleviate the sample-imbalance problem during the reconstruction process and realize the fusion of the centroid annotation and the focused vertebra. Furthermore, aiming at automatic and customized 3D spinal reconstruction in real-world scenarios, we extend ReVerteR to a clinical deployment-oriented framework, and develop an interactive interface with all functions in the framework integrated so as to enhance human–computer interaction during clinical decision-making. Extensive experiments and visualization conducted on our constructed datasets based on two benchmark datasets of spinal CT, VerSe 2019 and VerSe 2020, demonstrate the effectiveness of our proposed ReVerteR. In this paper, we propose an automatic 3D reconstruction method of vertebrae based on orthogonal bi-planar radiographs. With the 3D upright posture of the spine under natural weight bearing effectively constructed, our proposed method is expected to better support doctors make clinical decision during spine-related diagnosis and surgery.

## Introduction

Clinically, capturing the 3D models of spines is crucial for surgical planning, implant fitting, and postoperative evaluation^1^. However, existing 3D volumetric imaging modalities, i.e., magnetic resonance imaging (MRI) and computed tomography (CT) are not applicable for spine-related diagnosis and surgery under all the circumstances. First, regarding spine-related diagnosis, analysis of the spine’s shape and vertebral arrangement should be carried out in an upright posture under natural weight bearing, while the acquisition schemes of these 3D imaging modalities require the patient to be in a prone or supine position, which means that the patients must lie on their chest or back^2,3^. Second, regarding spine-related surgery, there are a series of spinal surgeries, e.g., scoliosis correction surgery, lumbar spondylolisthesis surgery, and lumbar spinal stenosis surgery, involved with high-risky screw-based fixation operations, where accurate 3D modeling of spinal posture is the necessary reference to avoid improper placement of spinal screws^4^. However, due to the limitation of the imaging equipment, it is infeasible to conduct MRI or CT imaging during the surgical process. Third, given the large radiation dose of CT and the prohibitive cost of CT and MRI, frequent imaging based on these 3D modalities is not appropriate from the perspective of either health or expense. Therefore, in view of the various problems when conducting CT and MRI on the real clinical scenarios, currently, 2D radiographs are actually the only choice for spinal modeling on many occasions. However, compared with 3D images, 2D radiographs lack enough spatial information and cannot provide doctors with intuitive features of spines from different views. To deal with the conflict between the limitations of existing 3D imaging modalities and the clinical requirements of 3D spinal modeling, research on computer-aided methods for 3D reconstruction of vertebrae based on 2D radiographs is expected to be an effective solution.

In the field of 3D reconstruction of spines, there have been a series of methods proposed^5–9^. For example, Pomero et al.^10^ and Humbert et al.^11^ proposed to infer the 3D vertebrae based on transversal and longitudinal information and determine the overall spine morphological information through part of parameters of vertebrae based on priori knowledge. However, these methods are too dependent on manual regulations and morphological databases, which is complicated and time-consuming.

In order to improve the automation of 3D reconstruction of the spine, Gajny et al.^12^ proposed an automatic corner detection method on the basis of transversal and longitudinal inference. The method first utilizes the database of spine parameters to estimate the 3D vertebral morphology, and then automatically detect lumbar and cervical vertebral corners and visible thoracic endplates. Finally, the performance was improved by manual rigid registration of the vertebrae. It can be seen that this method still requires manual intervention, which greatly limits the application scenario of the method and makes it difficult to provide intraoperative assistance.

Recently, with the development of deep learning algorithms, it has been feasible to further optimize the automation of reconstruction from 2D radiographs to 3D morphology of anatomical objects. For example, Yoni et al.^13^ successfully segmented the 3D structure of knee joint by utilizing the 3D U-Net architecture which fuses bi-planar radiographs. With the help of Convolutional Neural Networks (CNN), the method does not need manual operations and is thereby convenient and fast. However, different from the simple structure of the knee joint, the shape of the vertebra is much more complicated, which means that the effectiveness of neural network model on vertebral reconstruction still needs to be verified.

In terms of deep learning-based automatic 3D shape reconstruction of spines, Bayat et al.^14^ proposed TransVert. Based on a Fully Convolutional Network (FCN) architecture, TransVert can realize 3D reconstruction of spine morphology by utilizing digitally reconstructed radiographs (DRR) images from two perspectives: coronal and sagittal planes. TransVert is a representative method in the field of spine reconstruction. However, it is still difficult to verify the feasibility of TransVert, since the related experiments are conducted on non-open-source data, the designed framework of TransVert is simple, and the optimization purpose of TransVert is not clear. Additionally, Chen et al.^15^ also proposed a CNN-based encoder–decoder architecture BX2S-Net to reduce the semantic gap between feature maps and achieve information fusion for bi-planar X-ray- based 3D spinal reconstruction. However, they cropped vertebral-level images based on the annotation ground truth of CT images, which is impossible to conduct in clinical application stage and is not robust for noisy cases in real-word clinical scenarios.

In view of the relatively primitive and limited research status of automatic 3D reconstruction of spines, in this paper, we aim to propose a novel generative adversarial network (GAN) based framework, ReVerteR, which can realize automatic 3D Reconstruction of Vertebrae from orthogonal bi-planar Radiographs. The contributions of this work are summarized as follows:

- We propose a novel GAN-based architecture for fusing orthogonal radiographs to generate 3D shapes. with the utilization of ResUnet as the backbone of the generator, multi-scale information can be better obtained to make up for the lack of information in 3D vertebral reconstruction from two perspectives.
- We specially designed an integrated loss function in ReVerteR, which combines Dice, Hausdorff, Focal, and MSE to further alleviate the sample-imbalance problem during the 3D reconstruction process.
- We introduce self-attention mechanism into the architecture. Based on the Non-local network, ReVerteR can realize the information fusion of the centroid annotation and the focused vertebra.
- We extend ReVerteR to a clinical deployment-oriented framework for automatic and customized application in real-world scenarios and develop an interactive interface with all functions in the framework integrated to enhance human–computer interaction during clinical decision-making.
- Extensive experiments and visualization are conducted on two benchmark datasets of spinal CT, VerSe2019 and VerSe2020, which demonstrates the effectiveness of our proposed framework for 3D reconstruction of spines.

## Related work

### Medical imaging modalities

At present, there are three commonly used radiological diagnostic modalities in the field of spinal disease diagnosis: radiograph, CT, and MRI^16–19^. Although radiographs are more suitable for the diagnosis of spinal diseases than the other two modalities, they still have shortcomings due to their 2D form, e.g., lacking viewing angles, unintuitive, and insufficient in spatial information. CT images can provide rich 3D information, but they are not only with a high dose of radiation, but also expensive. Essentially, CT is the overlaying of 2D images, when doctors perform medical analysis, they take only one slice from the CT images which is still in 2D form. In addition, CT/MRI requires supine imaging, which may change the true curvature of the spine and is easy to result in misdiagnosis. In addition, the conduction of CT and MRI imaging is very limited, which means that intraoperative imaging is difficult. Therefore, the imaging of spines based on CT and MRI is quite limited.

### Medical 3D reconstruction methods

Regarding medical image reconstruction, the Department of Radiation Oncology, Stanford University^20^ first attempted to utilize a single-view radiograph to reconstruct CT images with a deep learning-based transformation module. Ying et al.^21^ proposed X2CT-GAN, which is the first work to explore CT reconstruction from bi-planar radiographs with a GAN-based architecture. Both works utilize deep learning methods to reconstruct CT images from radiographs and are not involved with the more challenging task, 3D reconstruction of the morphology of anatomical objects.

Regarding medical 3D reconstruction, Kasten et al.^22^ utilized an U-Net-based architecture to realize the end-to-end 3D reconstruction of the knee joint from bi-planar radiographs. In terms of 3D reconstruction of spine morphology, There have been also some studies conducted. However, most of these proposed methods are based on priori knowledge from morphological database and statistical models^10–12,23^, which means that doctors need to frequently intervene the inference of vertebral information. Recently, a FCN-based method TransVert was proposed, which utilized coronal and sagittal DRR images to reconstruct the 3D morphology of spines. However, the feasibility of TransVert is not verified. First, the experiments conducted on TransVert are based on the dataset whose ground truth is generated by a semantic segmentation model. the performance of the semantic segmentation model is important, since the structure of a vertebra is very complicated, the bad performance of semantic segmentation model will result in the information loss of the ground truth, which may overvalue the performance of the 3D reconstruction method. However, the semantic segmentation model utilized to generate ground truth for TransVert is not introduced in detail. In addition, the architecture design of TransVert is simple, it is expectable that the architecture can be further modified to improve the performance of 3D reconstruction.

### Medical 3D semantic segmentation

Regarding the semantic segmentation task, FCN^24^ is the first proposed architecture and has been the basic framework of semantic segmentation. Subsequent methods are all improved from this framework. Based on FCN, Ronneberger et al.^25^ proposed U-Net, which achieved impressive performance in the semantic segmentation for medical image.

Given that biomedical images usually need to present stereoscopic structures, e.g., CT. It is too complicated to use 2D segmentation methods to process 3D data. Therefore, on the basis of 2D Unet, 3D U-Net^26^, 3D ResUnet^27^, and Vnet^28^ are proposed to improve the performance of 3D semantic segmentation. Compared with 3D U-Net, 3D ResUnet and Vnet introduce the residual structure into the architecture, which further improves the training efficiency of 3D images. In this paper, 3D features and masks require to be mapped in the generator. Therefore, the 3D semantic segmentation architecture is also a key component in the proposed framework.

## Methods

**Figure 1:**
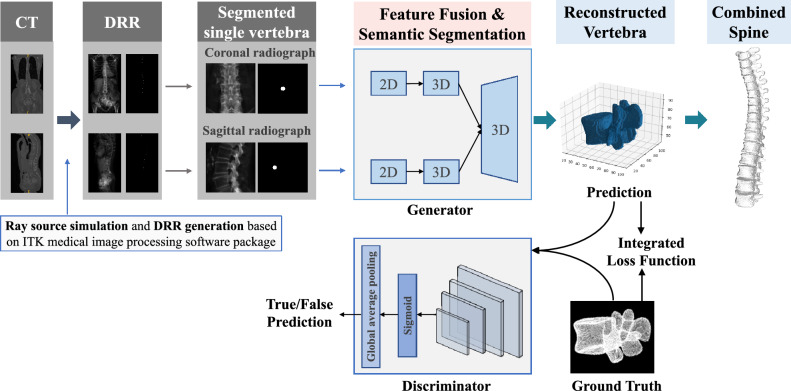
The overall processing workflow of our proposed ReVerteR.

In this section, we would introduce the design of our proposed framework ReVerteR in detail. The overall workflow of ReVerteR is shown in Fig. 1. ReVerteR is based on a GAN architecture, and thereby mainly consisting of two parts: a generator and a discriminator. The generator and the discriminator fight each other and adjust the parameters constantly, so that the discriminator network cannot judge whether the output of the generated network is true or not. Through the idea of game antagonism, we constantly optimize the performance of the generator to make it generate more realistic vertebrae. Therefore, the design of the generator is the core content of the whole model architecture, which includes 3 modules: (1) the centroid fusion module, (2) the 2D-3D transformation module, (3) and the fully convolutional segmentation network module.

**Figure 2:**
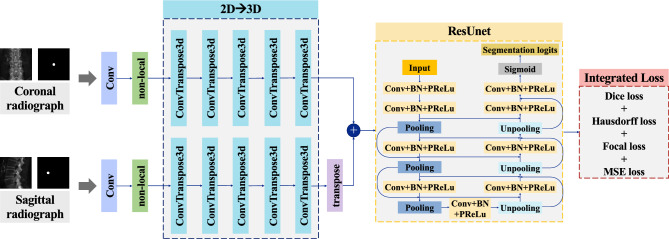
Architecture of ReVerteR, where the detailed design of the centroid fusion module and the 2D–3D transformation module are presented particularly.

Specifically, the centroid fusion module is designed to highlight coronal or sagittal radiographs in order to reconstruct the position of the vertebrae; The 2D–3D conversion module is mainly responsible for mapping coronal and sagittal 2D images from different perspectives to features in 3D space. The fully convolutional segmentation network module is designed make segmentation on the masked vertebral information from the 3D features based on supervised learning method. Detailed design of the modules in ReVerteR architecture is shown in Fig. 2.

### Centroid fusion module

**Figure 3:**
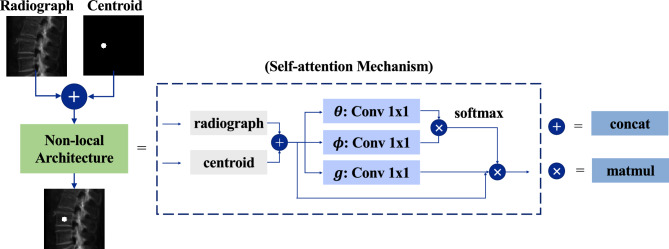
The fusion of centroid information and vertebral information under self-attention mechanism conducted by the Non-local architecture.

An adult spine is composed of 26 vertebrae, and the structure of each vertebra is complicated. If the spine is reconstructed as a whole structure, small components such as spinous process and transverse process of vertebrae are easily ignored. Meanwhile, a single radiograph contains a various information of bones, which means that it may also include redundant information such as ribs and leg bones. In order to better focus the model’s attention on the effect of vertebral reconstruction, each vertebra in the radiograph is segmented (cropped by 120 × 120 in width and height), which is like the radiograph input shown in Fig. 3. As a result, each patch is cropped for only one vertebra and may contain little redundant information from at most two adjacent vertebrae of the cropped object.

In order to make the model focus on the single vertebra expected to be reconstructed, the centroid mask is added. As shown in Fig. 3, the vertebrae expected to be reconstructed are annotated with centroid. Each patch pair with one centroid mask, the number of whom equals the number of vertebrae to be reconstructed. Concatenation operation is conducted to fuse the patch and the centroid information. Then, self-attention mechanism, which is conducted via Non-local blocks in Non-local Neural Networks^29^, is utilized to obtain the dependency relationship between non-adjacent pixels in the image, so as to fully integrate centroid information and vertebral information. Detailed architecture of Non-local is shown in Fig. 2 and the formula is shown below:

1 $$output=x+{\upalpha}\left(softmax\left(\uptheta {\left(x\right)}^{T}\upphi \left(x\right)\right)\upsigma {\left(x\right)}^{T}\right)$$

where θ(x), φ(x), σ(x) denote the 1 × 1 convolution operations conducted on the feature *x*, respectively, which transforms feature map to one dimension. Then, the dot production is conducted between θ (x) and φ (x), and softmax function is utilized for normalization, so as to obtain the similarity between each point on the feature map and all other pixels. Finally, the weight is product with σ (x) and the output is obtained.

### 2D–3D transformation module

The main purpose of the 2D-3D transformation module is to extend 2D features into 3D features. The difference between 2D images and 3D images is that 2D images lack depth information. Therefore, we duplicate the 2D images and pile them together to obtain the 3D information.

First, each view (lateral and anterior) is duplicated 120 times in one dimension. That is, we expand the lateral view on the coronal axis, and expand the frontal view on the sagittal axis. Second, given the antero-posterior and latera angle information, the sagittal features are rotated with the coronal plane as the main direction. Finally, the coronal and sagittal planes are fused by conducting concatenation operation.

### Fully convolutional segmentation network module

Fully convolutional segmentation network module is designed as the process from 3D features to 3D vertebra masks, which can be regarded as a 3D semantic segmentation task. In this module, our designed ReVerteR utilize 3D ResUnet architecture^27^ as the main backbone network. Through supervised learning, each individual voxel is classified, and the vertebral information is annotated from the features in shape of 120 × 120 × 120. ResUnet introduces the residual module and the Batch Normalization layer on the basis U-Net network, in which way to construct deeper network structure and avoid vanishing gradient. Meanwhile, 2D images are directly input into the ResUnet model after dimension amplification without reshaping operations were performed, in which way to retain the information from each view as much as possible. The skip-connection module in ResUnet is utilized to integrate multi-scale and multi-level semantic information, which can provide richer information for vertebral segmentation.

In general, the generator in ReVerteR utilizes 3D ResUnet as the main backbone network. With the introduction of self-attention mechanism, the complementary information from different perspectives can be fused based on centroid annotation information, and 2D features can then be transformed into 3D features. Finally, 3D vertebra mask is utilized for supervised learning of 3D features to realize the reconstruction task from 2D image to 3D morphology. Meanwhile, the discriminator in ReVerteR is mainly based on PatchGAN^30^, which can focus more on the localized impact in the form of full convolution. Specifically, in a PatchGAN, the discriminator network is designed to classify individual patches (small regions) of an image as real or fake, rather than classifying the entire image. This approach allows for more localized and fine-grained feedback to the generator network during training, which can lead to improved image quality and detail preservation in the generated images.

### Design of integrated loss function

In addition to a MSE-based adversarial loss, we propose an integrated loss function which mainly combines Dice loss, Hausdorff loss, and Focal loss as the optimization objective. We set the hyper parameter λ, α, β, and γ to adjust the weight of Dice loss, Hausdorff loss, Focal loss and MSE loss in the integrated loss function, respectively. Dice loss was proposed in V-Net^28^ and has been widely applied in medical image segmentation. When 3D vertebrae are segmented, the focused structure of the vertebrae occupies only a very small area in the shape of 120 × 120 × 120. If the proportion of target voxels is too small, the learning process will easily fall into the localized minimum of the loss function. Therefore, it is necessary to optimize the optimization objective reasonably and increase the weight of the foreground area. Dice loss is a region-related loss function. The intersection and union between the prediction and the mask are calculated, so that the loss error is only related to the foreground target. Although Dice loss can better deal with the issue of unbalanced positive and negative samples, training loss is prone to instability. Therefore, we further introduce two other metrics, Hausdorff loss and Focal loss into the integrated loss, considering their significance for vertebra reconstruction from different perspectives. In this paper, λ is set as 10, α is set as 0.0001, β is set as 10, and γ is set as 0.1 for ReVerteR. The integrated loss function of this model is as follows:

2 $$\mathcal{L}\left(G,D\right)=\lambda {\mathcal{L}}_{Dice}\left(G\right)+{\upalpha}{\mathcal{L}}_{\text{Hausdorff}}\left(G\right)+\beta {\mathcal{L}}_{\text{Focal}}\left(G\right)+\gamma {\mathcal{L}}_{GAN}\left(G,D\right)$$

where λ, α, β, and γ denotes the hyper parameters we set to adjust the weight of the losses. ${\mathcal{L}}_{GAN}(G,D)$ consists of the generator *G* and the discriminator *D*:

3 $${\mathcal{L}}_{GAN}\left(G\right)={E}_{x\sim {p}_{Xpredict}}{\left(D\left(G\left(x\right)\right)-1\right)}^{2}$$

4 $${\mathcal{L}}_{GAN}\left(D\right)={E}_{y\sim {p}_{Ymask}}{\left(D\left(y\right)-1\right)}^{2}+{E}_{x\sim {p}_{Xpredict}}{\left(D\left(G\left(x\right)\right)-0\right)}^{2}$$

where *x* denotes the corresponding centroid information of the radiograph or the DRR image, and *y* denotes the vertebral morphology we expect to reconstruct.

5 $${\mathcal{L}}_{Dice}\left(G\right)=1-Dice=1-\frac{2\left|{X}_{predict}\cap {Y}_{mask}\right|}{\left|{X}_{predict}\right|+\left|{Y}_{mask}\right|}$$

where *Y*_*predict*_ denotes the prediction result, which is also the output of the framework. *Y*_*mask*_ denotes the vertebral morphology we expect to reconstruct.

6 $${\mathcal{L}}_{Hausdorff}\left(G\right)=Los{s}_{\text{DT}}\left(q,p\right)=\frac{1}{\left|\Omega \right|}{\sum }_{\Omega }\left({\left(p-q\right)}^{2}\circ \left({d}_{p}^{\upalpha}+{d}_{q}^{\upalpha}\right)\right)$$

where *p* denotes the predicted result, *q* denotes the ground truth, and *dp* denotes the distance from the predicted segmentation result to the boundary.

7 $${\mathcal{L}}_{Focal}\left(G\right)=-{\upalpha}_{t}{\left(1-{p}_{t}\right)}^{\upgamma }\text{log}\left({p}_{t}\right)$$

where *pt* denotes the probability of prediction as a positive sample, α*t* is set as 0.2, and γ is set as 5.

### Clinical deployment-oriented automatic framework & interactive interface

Aiming at automatic clinical deployment of our proposed 3D reconstruction method in real-world scenarios. We further extend ReVerteR to a whole framework which integrates both ReVerteR as the 3D reconstruction module and an automatic centroid annotation module inspired by our previous work MPF-net^31^.

Regarding the design of MPF-net, it is a CNN-based multi-task framework which focuses on both anterior–posterior (AP) view X-rays and lateral (LAT) view X-rays, conducting joint learning of vertebra detection and landmark prediction. With information from neighbor vertebra and bi-planar X-rays get fully integrated. MPF-net can realize effective and automatic detection on the four key landmarks on each vertebra, and thereby being further utilized to generate the centroid mask for each vertebra.

**Figure 4:**
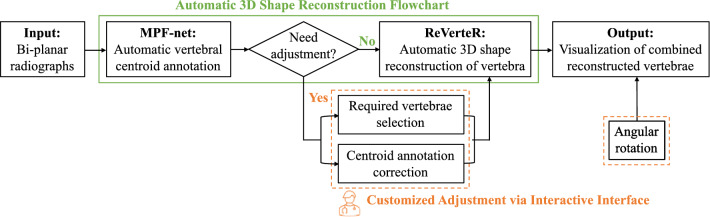
Flowchart of the whole framework for automatic 3D reconstruction of vertebra designed based on ReVerteR for clinical deployment.

The whole framework flowchart is shown in Fig. 4 below. Specifically, all bi-planar radiographs will first be automatically annotated with centroid masks based on the MPF-net-based architecture, and then directly perform automatic 3D shape reconstruction based on ReVerteR if no further adjustment required. Therefore, the whole 3D shape reconstruction process can be conducted in an automatic approach. Furthermore, aiming to enhance the interactive between the 3D reconstruction system and clinicians so as to facilitate clinical decision-making, we design two parts of customized adjustment functions based on the automatic system. First, based on the automatic centroid annotation results, clinicians can choose whether get involved (a) to choose the vertebrae required for reconstruction, and (b) to manually correct centroid masks if they do not agree with the model-based annotation result. Second, clinician can conduct angular rotation of the visualization results of vertebral 3D shape reconstructed by ReVerteR. Consequently, the vertebrae can be observed from any different views according to clinicians’ preference.

**Figure 5:**
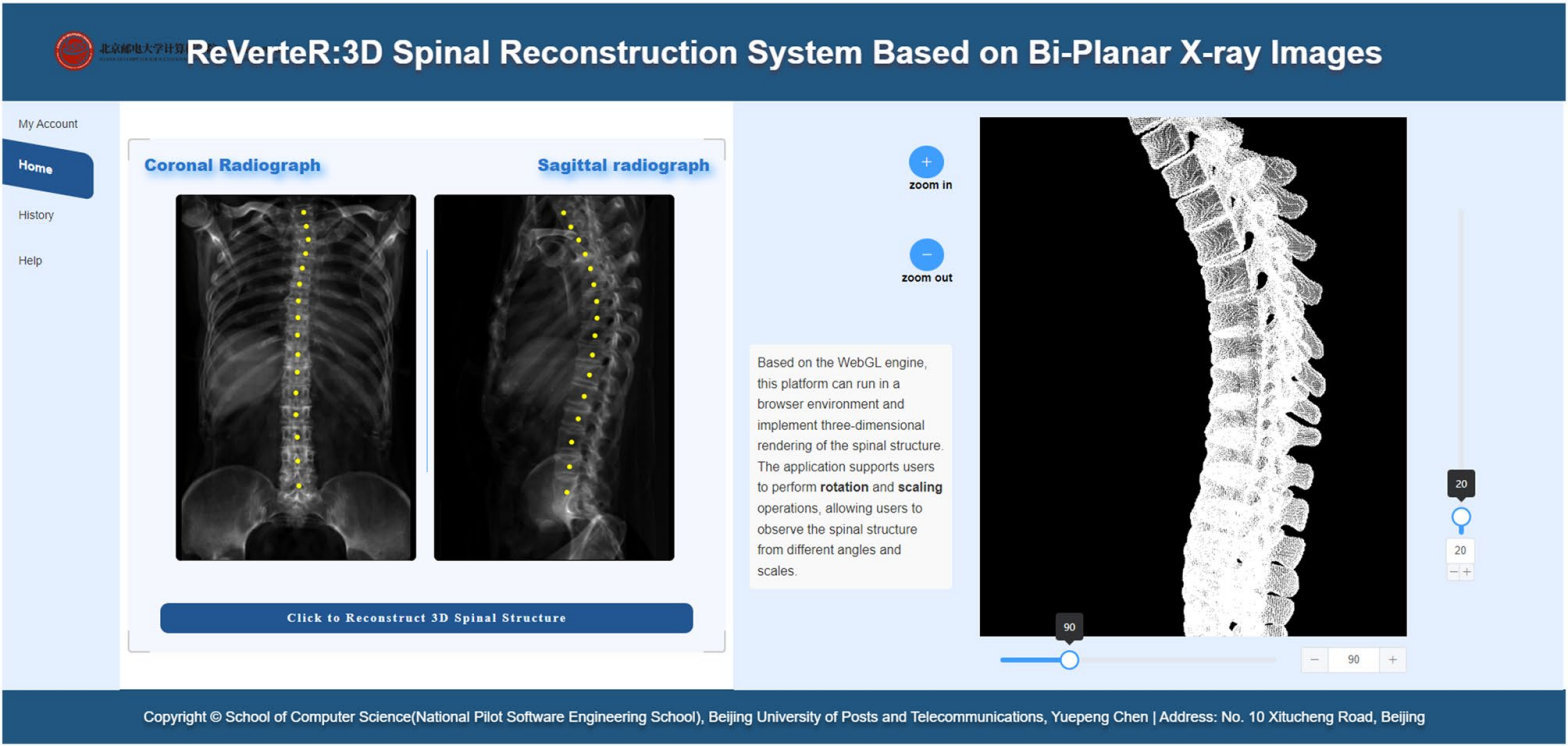
Interactive interface developed based on our proposed clinical deployment-oriented 3D spinal reconstruction framework, where automatically centroid-annotated bi-planar radiographs and reconstructed 3D shape are both presented. A series of customized functions are also integrated.

Additionally, we have also developed an interactive interface based on the clinical deployment-oriented framework as is shown in Fig. 5. It can be seen that all customized adjustment functions are integrated in the interface. Specifically, the input bi-planar radiographs are presented on the left side, where automatically annotated centroid masks are marked and can be removed or corrected. Here we simply use two centroid-annotated DRRs to simulate the presentation of bi-planar radiographs in the interface. The output reconstructed 3D vertebrae or spine is presented on the right side, where the view of the visualization result can be adjusted by the sliding button. To our best knowledge, this is the first study which proposes a clinical deployment-oriented 3D vertebral reconstruction framework and develop an interactive interface for the reconstruction system.

### Ethical approval

This project is waived from ethical approval and only involves data from two publicly available datasets VerSe 2019 and VerSe 2020 for the validation.

## Results

In this section, we would conduct a series of experiments, including performance comparison, ablation study, and visualization, on the two benchmark datasets of spinal CT, VerSe2019 and VerSe2020, to demonstrate the effectiveness of our proposed ReVerteR.

### Dataset

Ideally, to train and validate our proposed vertebral reconstruction method, we would need a large database of paired radiographs and their corresponding vertebral masks. However, there is currently no public paired spine dataset, and there are rarely two medical images taken at the same time clinically. Therefore, we utilize the two publicly available spine segmentation datasets, VerSe2019 and VerSe2020^32^, which provide spine CT data and corresponding spine 3D mask annotation, and construct two versions of datasets VerSe-full and VerSe-small.

In terms of VerSe-full we simply combine all the vertebrae datasets from VerSe19 and VerSe20, which contains 374 spines and 4522 vertebrae in total. In terms of VerSe-small, aiming to maintain the balance of distribution of different types of vertebrae and the consistency of vertebrae in a spine, we further screen samples from VerSe-full in units of spine following 3 constraints. Specifically, we would filter out the whole spine-level sample if it contains (a) a T13, L6 or cervical vertebra; (b) no more than 2 thoracic vertebrae; and (c) metal occlusion (e.g., pedicle nail occlusion). As a result our constructed VerSe-small dataset contains 140 spines and 1407 vertebrae.

**Figure 6:**
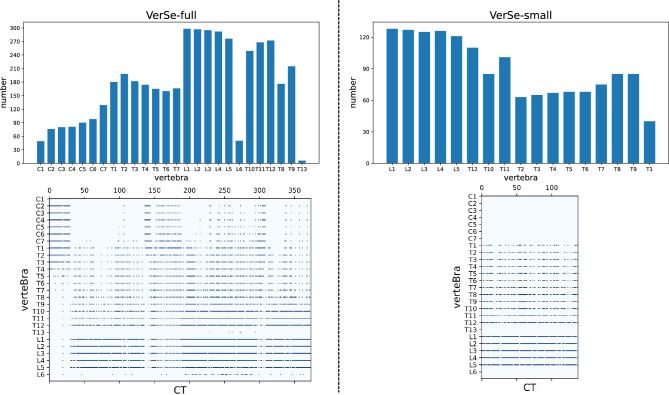
Histograms and matrix plots which of the sample distribution of different types of vertebrae in the datasets VerSe-full and VerSe-small.

The statistics of VerSe-full and VerSe-small are shown in Table 1. Detailed distribution of different types of vertebral samples in the two datasets are shown in the histograms and matrix plots in Fig. 6. It can be seen that the vertebra samples screened in VerSe-small are much more densely, evenly, and consistently distributed in each spine samples.

**Table 1:** Statistics of our constructed datasets VerSe-full and VerSe-small.

	VerSe-full	VerSe-small
Number of subjects	355	140
Number of spines	374	140
Number of vertebrae	4522	1407

**Figure 7:**
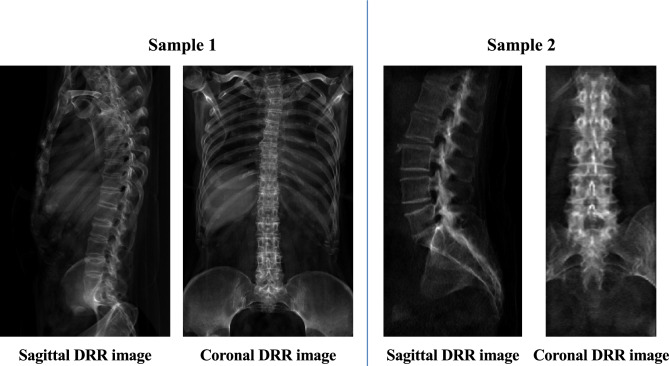
Samples of paired DRR images.

We integrate the images in both datasets and there by generating a whole dataset. The corresponding bi-planar radiographs are synthesized by conducting digital reconstruction radiography (DRR) technology^33^. Figure 7 presents the synthesized samples based on DRR technology.

DRR is a method used to simulate the projection of 3D reconstructed images from a specific direction or from the direction of an X-ray target similar to that of a simulation positioning device. This method finds wide applications in CT simulation positioning, Image-Guided Radiation Therapy (IGRT), and computer-assisted surgery, among others. Our DRR reconstruction algorithm primarily employs X-ray casting techniques. Specifically, we first input the 3D image data into our algorithm. Subsequently, our algorithm simulates the passage of X-rays through this 3D image from a particular direction, recording the intensity of X-rays passing through each pixel. Finally, we map these intensity values onto a 2D plane, resulting in a 2D radiographic image. Regarding cropping of DRR images, we first perform DRR on the original 3D CT images, and then crop the DRR images for each vertebra based on the cropping box with centroid as the center.

### Experimental settings

The proposed framework is implemented with Pytorch platform on a machine equipped with an Intel Xeon Gold 6230 CPU and NVIDIA GeForce RTX 4090 GPU, the generator and the discriminator are trained alternately. In our experiment, the epoch number is set as 100, the batch size is set as 8, the learning rate is initially set as 0.0001, decaying to one-tenth of its original value every 20 epochs, and the input image size is set as 120. In both two datasets, we split samples for the training set and test set in units of patient with the ratio set as 4:1. All methods are trained using the Adam optimizer, and are parallelly computed on two GPUs. The input size of the coronal and sagittal vertebral images is set to 120 × 120, and the output size of the methods is set to 120 × 120 × 120. For baseline methods in comparative experiments, the input and output dimensions of the model are kept consistent with our method, while the remaining parameters are set according to the parameter settings in their paper. In the experiments, we run all the models 10 times and report the mean value of them so as to avoid bias because of randomization.

### Evaluation metrics

To better evaluate the effectiveness of our proposed method, being consistent with previous domain-related work, in this study, we utilize Dice value, the 95th percentile Hausdorff distance (HD95), and the Normalized Surface Distance (NSD) as the evaluation metrics to reflect the effect of 3D vertebral morphology reconstruction.

Dice: Dice is the most frequently used metric in medical image segmentation tasks. As a set similarity metric, Dice is designed to calculate the similarity of two samples, and thereby can be utilized to evaluate the overlap rate between our reconstructed result and ground truth spinal morphology. The value threshold is [0, 1], where the best segmentation result is 1, while the worst segmentation result is 0.

HD95: Hausdorff distance is a measure that describes the degree of similarity between two sets of points. Dice is sensitive to the internal region of mask, while Hausdorff distance is sensitive to the segmented boundary, which corresponds to the complex anatomy of vertebrae and can reflect the segmentation effect on morphology. HD95 capture the extent of variation in distance values while also being less sensitive to outliers compared to the traditional Hausdorff distance. A higher HD95 value indicates greater dissimilarity between the two sets of points, while a lower value suggests greater similarity.

NSD: NSD measures the average distance between corresponding points on the reconstructed surface and the reference surface, normalized by a characteristic length of the object being reconstructed. NSD is a dimensionless metric that ranges from 0 to 1. A higher NSD value indicates better alignment and agreement between the reconstructed and reference surfaces.

### Comparative experiment

To verify the effectiveness of our proposed ReVerteR, we conduct extensive comparative experiments on 8 domain-related representative baseline methods which can generally be decomposed into 4 types as listed below. (a) TransVert^14^, BX2S-Net^15^, and an Unet-based method^13^ which are all CNN-based methods designed for the task of 3D shape reconstruction of bones, e.g., vertebra and patella. (b) SwinUNETR^34^, UNETR^35^, and AttentionUnet^36^ which are CNN or transformer-based methods designed for the task of medical image segmentation, e.g., MRI and CT. (c) OneDConcat^37^ which is spinal reconstruction method designed based on Electro-Optical System (EOS) imaging technology. (d) X2CT-GAN-inspired method^21,38^ which are CNN-based methods designed for the task of the reconstruction of CT images. In terms of the baselines that are not originally designed for the task of 3D shape reconstruction, we modify their architectures by adding the 2D–3D feature fusion module or the 3D image segmentation architecture, respectively, as designed in our proposed method ReVerteR. Detailed results of the comparative experiments in two datasets VerSe-full and VerSe-small are listed in the Table 2. It can be seen that our proposed method ReVerteR outperforms all the representative baselines on two the datasets only except the X2CT-GAN-inspired method in terms of the metric HD95 in the dataset VerSe-full, which demonstrates the effectiveness of our method regarding the task of 3d vertebrae reconstruction from orthogonal bi-planar radiographs.

**Table 2:** Performance of our proposed ReVerteR and the representative baseline methods. We run all models 10 times and report the mean value of Dice value, the 95th percentile Hausdorff distance (HD95), and the Normalized Surface Distance (NSD) as the evaluation metrics. The symbol ‘+’ and ‘−’ indicate that the higher and lower the value is, the better the model performs, respectively. Best results are marked bold.

	VerSe-full			VerSe-small		
	Dice (+)	HD95 (−)	NSD (+)	Dice (+)	HD95 (−)	NSD (+)
TransVert ^1^	0.7743	5.4480	0.6718	0.7647	6.6468	0.6094
BX2S-Net ^2^	0.7558	6.2984	0.6345	0.7494	7.5000	0.5824
Unet ^3^	0.7712	5.5935	0.6691	0.7582	6.7097	0.6160
SwinUNETR ^4^	0.7759	5.2873	0.6821	0.7531	6.9923	0.5964
UNETR ^5^	0.7584	5.9160	0.6546	0.7421	6.7891	0.6012
AttentionUnet ^6^	0.7696	5.6788	0.6660	0.7535	6.8888	0.6085
OneDConcat ^7^	0.7822	5.0832	0.6904	0.7637	6.6105	0.6106
X2CT-GAN ^8,9^	0.7870	**4.4931**	0.7136	0.7600	6.5289	0.6073
ReVerteR (Ours)	**0.7938**	4.5836	**0.7182**	**0.7685**	**6.3129**	**0.6198**

Specifically, our proposed ReVerteR outperforms TransVert and BX2S-Net which are the only two methods originally designed for the task of 3D shape reconstruction of vertebrae. Neither of the two methods performs as reported in their paper, which result from different dataset settings in the experiment. First, in the experiments in their original paper, both methods involve extra noisy labelled datasets apart from the VerSe dataset. The training set of TransVert is augmented by involving 954 X-ray images annotated by themselves based on a pre-trained image segmentation model, while the dataset of BX2S-Net is augmented with 1000 noisy-labelled X-rays from dataset CTSpine1K^39^. Such data augmentation may lead to noise and biases during model training. Second, in the experiments of BX2S-Net, the vertebra-level patches are cropped based on ground truth of CT images and thereby getting rid of redundant information of adjacent vertebrae. However, such patch preprocessing measure is impossible to conduct and not robust for cases in real-word clinical scenarios.

**Figure 8:**
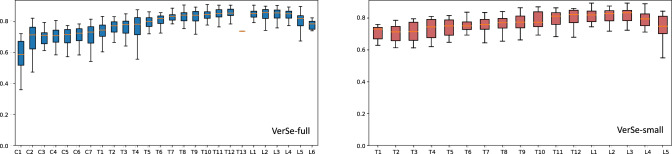
Box plots of Dice metric, which present the position-specific performance of ReVerteR on VerSe-full and VerSe-small, respectively.

Furthermore, we conduct a more fine-grained analysis on our proposed ReVerteR. Figure 8 presents the position-specific Dice performance of ReVerteR on VerSe-full and VerSe-small. It can be seen that our proposed method overall performs robustly on all positions of vertebrae. However, a relative performance gap can be observed on the cervical vertebrae compared with lumbar and thoracic vertebrae, which should be reasoned for the relatively lacking annotated samples in the datasets, which is alleviated in VerSe-small, as well as the its tiny morphology, which makes the 3D shape reconstruction of cervical vertebrae a more difficult task.

### Ablation study

We also conduct a series of ablation study to demonstrate the effectiveness of a series of architectures and strategies designed in our proposed method ReVerteR, including the centroid fusion module, the non-local architecture, the Dice loss, the Focal + Hausdorff loss function, and the adversarial learning architecture. We tested different without (w/o) settings to separately remove each key architecture and compare them with the original setting. As is shown in Table 3, in terms of the design of loss function, compared with our designed integrated loss function, the Dice value of the function without Dice loss drops to 0.4991 and 0.4574, while the Hausdorff distance significantly increase to 13 and 11. Such huge model performance deteriorate demonstrates the key effect of the Dice loss. Given that a vertebra only occupies a small region in a patch, Dice loss can help focus more on the targeted voxels by enlarging the attention to foreground samples, which significantly improves the reconstruction performance. Additionally, Focal loss and Hausdorff loss also contribute to better performance of our proposed method, which demonstrate the integrated loss functions are complementary to each other when capturing the features during the 3D reconstruction. The Non-local architecture utilized in the stage of 2D-to-3D transformation does effectively fuse the features from two perspectives of radiographs. Meanwhile, in terms of the centroid fusion module since multiple vertebrae may simultaneously appear in a single patch image. the fusion of centroid information is expected to provide the priori knowledge of the targeted vertebrae we’d like to reconstruct. The ablation results shown in Table 3 do demonstrate our hypothesis that the introduction of centroid annotation can help the method focus more on the targeted vertebra. Last but not least, it can be seen that the adoption of the adversarial learning architecture does have a positive effect on the performance of 3D vertebra reconstruction from all the three evaluation metrics.

**Table 3:** Ablation study on the design of loss function, centroid fusion module, and adversarial learning strategy. We run all models 10 times and report the mean value of Dice value, the 95th percentile Hausdorff distance (HD95), and the Normalized Surface Distance (NSD).

	VerSe-full			VerSe-small		
	Dice (+)	HD95 (−)	NSD (+)	Dice (+)	HD95 (−)	NSD (+)
Fusion of centroid (w/o)	0.7774	5.2345	0.6703	0.7512	7.3896	0.5702
Non-local (w/o)	0.7800	5.3749	0.6763	0.7492	7.8278	0.5681
Dice loss (w/o)	0.4991	13.7402	0.3261	0.4574	11.5412	0.4144
Focal loss + Hausdorff loss (w/o)	0.7852	5.00988	0.6896	0.7557	7.1701	0.5823
adversarial learning (w/o)	0.7859	5.0647	0.6896	0.7559	7.3753	0.5802
ReVerteR (Ours)	0.7938	4.5836	0.7182	0.7685	6.3129	0.6198

### Statistics of implementation time

Our proposed method conducts 3D shape reconstruction of spine in units of vertebra, which means that clinicians can flexibly select which part of the spine needs to be reconstructed and visualized. To quantitatively present the implementation time for the reconstruction of the whole spine (26 vertebrae), here we make statistics of the average reconstruction time for each position of vertebra and the overall spine. We tested our proposed ReVerteR on VerSe-full dataset. As is shown in Table 4, our proposed method is efficient for different types of vertebra reconstruction and the overall average reconstruction time for the whole spine is 7.7204 s, which is efficient and usable for clinical deployments.

**Table 4:** Statistics of the average implementation time (in s) of ReVerteR for different positions of vertebrae tested on VerSe-full.

Position	C1	C2	C3	C4	C5	C6	C7	T1	T2
Time	0.0756	0.0739	0.0732	0.0819	0.0954	0.0783	0.0782	0.0765	0.0758
Position	T3	T4	T5	T6	T7	T8	T9	T10	T11
Time	0.0772	0.0789	0.0772	0.0777	0.0753	0.0766	0.0785	0.0785	0.0785
Position	T12	T13	L1	L2	L3	L4	L5	L6	Spine
Time	0.0780	0.0838	0.0788	0.0796	0.0775	0.0801	0.0770	0.0849	7.7204

### Visualization

**Figure 9:**
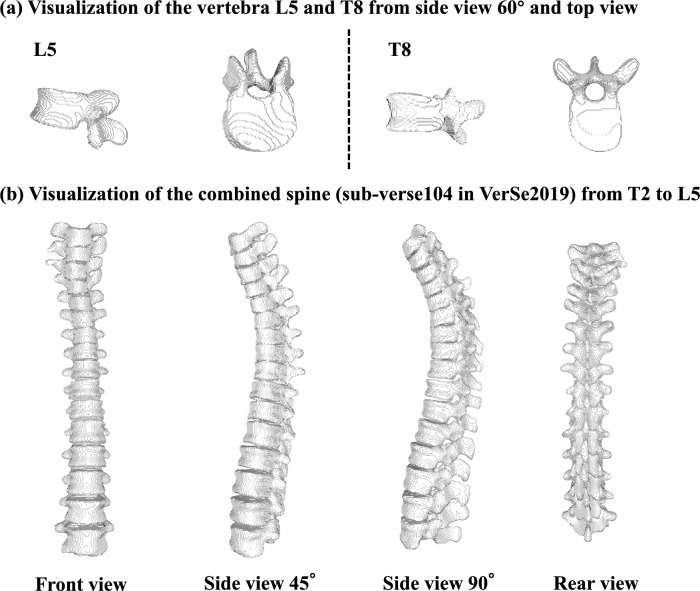
Visualization of the 3D reconstruction results of ReVerteR, where samples of both reconstructed vertebra and further combined spine are presented from different views.

Figure 9 shows the visualization results of 3D shape reconstruction of single vertebra samples and combine spine samples from different views based on Python, respectively. It can be seen that our method not only reconstructed the overall structure of the vertebral body, but also well reconstruct the complicated small components such as spinous process, transverse process and superior articular process. Additionally, the adjacent vertebrae can fit each other as is presented in the spine under natural weight bearing. Such variable visualized reconstruction results of vertebrae and spines from different views are accessible by deploying our developed interactive interface where the reconstructed object can be rotated with customized angles and directions.

## Discussion

This study focuses on the 3D shape reconstruction of spine based on bi-planar radiographs, proposing a novel method ReVerteR, which effectively optimizes the clinical decision-making requirement of 3D upright posture of the spine under natural weight bearing 3D. Specifically, we propose a self-attention mechanism- based centroid fusion module, which adopts non-local architecture to realize cross-channel information fusion of the centroid annotation and the focused vertebra, thereby effectively leveraging the vertebra-related knowledge while getting rid of the redundant information in X-ray images. Then, the 2D–3D feature fusion module is proposed to integrate the sagittal and coronal features through an orthogonal approach and get deeply fused with a ResUnet-based architecture. Furthermore, an integrated loss function which combines Dice loss, Hausdorff loss, Focal loss, and MSE loss is designed to optimize the training so as to better fit vertebral 3D morphology. Last but not least, a complete framework which consists of automatic centroid annotation and 3D reconstruction based on ReVerteR is designed for automatic clinical deployment of the system. An interactive interface is also developed based on ReVerteR to provide customized and visualized clinical assistance. To the best of our knowledge, this is the first study which proposes a clinical deployment-oriented 3D vertebral reconstruction framework and develop an interactive interface for the reconstruction system. Extensive comparative experiments and ablation study have been conducted based on our constructed two versions of VerSe datasets demonstrate the effectiveness of both the overall 3D reconstruction method and a series of architectures and strategies designed in the framework.

In future work, further study can be conducted to optimize our proposed ReVerteR and the developed interface. First, although we have involved knowledge of centroid mask into the method, more landmarks and more fine-grained relative coordinate can be involved as calibration information of the 3D environment, which is expected to improve the performance of 3D shape reconstruction. Second, regarding the clinical deployment of the 3D reconstruction system, although we have designed a framework to achieve full automation, given the complex real-world clinical decision-making scenarios, more efforts should be put into the optimization of collaboration between 3D reconstruction method and clinicians. With the aim to balance the accuracy and efficiency of decision-making, human-in-the-loop learning framework thereby should be treated as a potential solution to further optimize the human–computer interaction of the 3D reconstruction system. Third, given that lacking real-world bi-planar radiographs, in this work, we adopt DRR to simulate the multi-view radiograph inputs, which, however, would cause biases due to style transferring issue. Therefore, in future work, collection of real-world bi-planar radiographs should be a significant requirement to put forward. Additionally, transfer learning based on real-world radiograph datasets would be a promising approach to improving the robustness and the adaptability of the 3D reconstruction method in clinical scenarios.

## Conclusion

Aiming to deal with the conflict between the limitations of existing 3D imaging modalities and the clinical requirements of 3D spinal modeling, in this paper, we propose ReVerteR, a novel GAN-based framework to realize automatic 3D reconstruction of vertebras from orthogonal bi-planar radiographs. Based on supervised learning on DRR images, ReVerteR can fuse the vertebral information from 2D sagittal and coronal radiographs and the centroid information from the annotations. Extensive experiments conducted on the benchmark datasets demonstrate the effectiveness and rationality of our designed framework. In the future work, we will further modify the for the real-world clinical deployment to assist spine-related analysis.

## Data Availability

The datasets generated and analysed during the current study are available in the VerSe2019 repository, https://osf.io/nqjyw/ and the VerSe2020 repository, https://osf.io/t98fz/.

## Code availability

The source code of the project will be accessible on the Github link: https://github.com/chenchenchenyue/ReVerteR.
